# An Unusual Case of Bilateral Lower Extremity Edema in the Elderly: Immunoglobulin A (IgA) Vasculitis

**DOI:** 10.7759/cureus.42684

**Published:** 2023-07-30

**Authors:** Toyoshi Yanagihara, Takuya Nakagawa, Haruko Nishie, Yuki Moriuchi, Hiroaki Ogata, Masako Kadowaki, Atushi Moriwaki, Makoto Yoshida

**Affiliations:** 1 Department of Respiratory Medicine, National Hospital Organization Fukuoka National Hospital, Fukuoka, JPN; 2 Department of Dermatology, National Hospital Organization Fukuoka National Hospital, Fukuoka, JPN

**Keywords:** rash, leukocytoclastic vasculitis (lcv), skin lesion biopsy, lower extremity edema, iga vasculitis

## Abstract

We report a case of a 74-year-old male who exhibited bilateral lower extremity edema over three days. Examination revealed no signs of heart, renal, or hepatic failure, and hypothyroidism was also ruled out. An outpatient regimen of 40 mg furosemide was initiated. At a 12-day follow-up, although the edema had improved, the patient had developed pain in both lower limbs, especially ankles, accompanied by numerous petechiae and erythemas, some of which had formed papules. Skin biopsy of the rash displayed leukocytoclastic vasculitis with immunoglobulin A (IgA) deposition within the vascular walls, leading to a diagnosis of IgA vasculitis. Given the rarity of IgA vasculitis in elderly patients and the broad spectrum of potential diagnoses related to bilateral lower extremity edema in this population, IgA vasculitis can be easily overlooked. While this case did not present with glomerulonephritis, regular renal function monitoring is recommended due to the prognostic implications of renal involvement in adult-onset IgA vasculitis.

## Introduction

Immunoglobulin A (IgA) vasculitis, formerly known as ﻿Henoch-Schönlein purpura, is a form of small vessel vasculitis characterized by the deposition of IgA1 antibodies predominantly at the affected vessel walls [1,2]. It can manifest as either systemic or limited vasculitis affecting a single organ. Commonly involved sites include the skin, kidneys, gastrointestinal tract, and joints [1]. IgA vasculitis primarily occurs in children, making it the most prevalent systemic vasculitis in this age group [2]. Moreover, its occurrence in adults is uncommon, with incidence rates between 0.1 and 1.8 per 100,000 adults [3]. Unlike its pediatric counterpart, IgA vasculitis that emerges during adulthood presents a more complex clinical trajectory, higher recurrence rates, and poorer kidney outcomes [4]. Consequently, the full spectrum of clinical symptoms in adults remains inadequately defined. Here, we report a case of IgA vasculitis in the elderly with its initial symptom of bilateral lower extremity edema, which can be misdiagnosed due to various differential diagnoses.

## Case presentation

A 74-year-old male patient presented with bilateral lower extremity edema and a weight gain of 5 kg over three days. This onset followed a week after the patient experienced common cold symptoms, including nasal discharge, cough, and phlegm. The patient's medical history was bronchial asthma, sinusitis, hypertension, type 2 diabetes, and dyslipidemia, all well managed. No new medications had been initiated prior to the emergence of symptoms.

At initial examination, the patient had no complaints of dyspnea, with an oxygen saturation (SpO_2_) of 95%, a body temperature of 36.5℃, and a heart rate of 55 beats per minute. The chest examination revealed no abnormal sounds (rales), but bilateral lower extremity edema was present. The patient did not exhibit any symptoms or signs pertaining to gastrointestinal tract anomalies. The blood test results were as follows: white blood cell (WBC) count 6,480/µL, platelet count 179×10^3^/µL, C-reactive protein (CRP) 0.61 mg/dL, lactate dehydrogenase (LDH) 241 U/L, creatine kinase (CK) 284 U/L, blood urea nitrogen (BUN) 13.4 mg/dL, creatinine (Cre) 0.86 mg/dL, estimated glomerular filtration rate (eGFR) 66 ml/min, total cholesterol (T-Chol) 165 mg/dL, triglycerides (TG) 200 mg/dL, sodium (Na) 142 mmol/L, potassium (K) 3.8 mmol/L, chloride (Cl) 103 mmol/L, NT-proBNP 227 pg/mL, thyroid-stimulating hormone (TSH) 5.4 uIU/mL, and free T4 0.86 ng/mL. A chest X-ray did not reveal any cardiomegaly or signs of pulmonary edema. Outpatient treatment was initiated with 40 mg furosemide..

**Figure 1:**
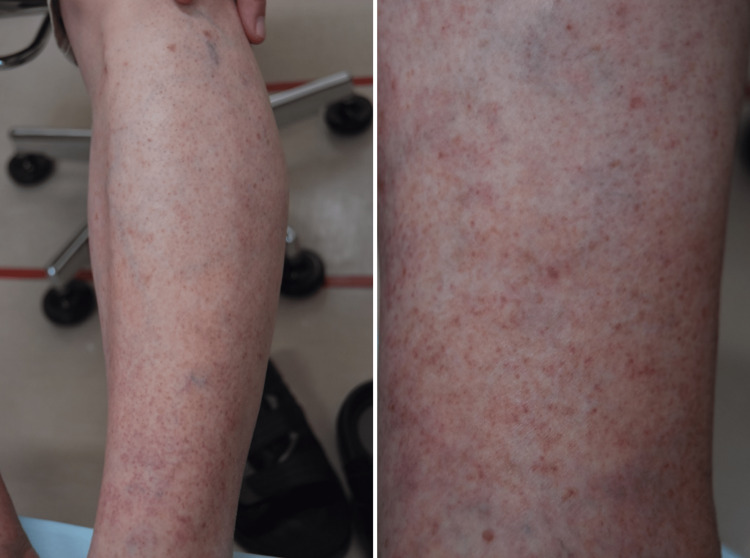
Lower extremities show numerous petechiae and erythemas, with some forming papules.

At a 12-day follow-up, although the edema had improved, the patient developed pain in both lower extremities, especially the ankles. This was accompanied by numerous petechiae and erythemas, with some forming papules (Figure 1).

**Figure 2:**
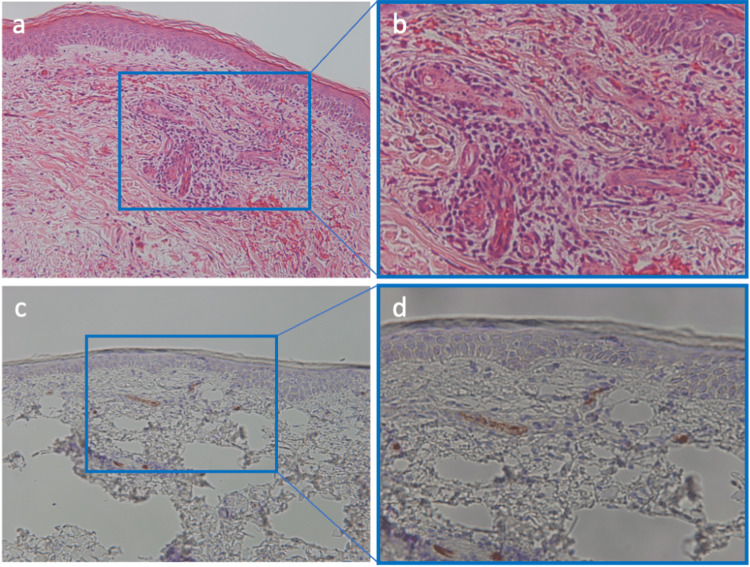
Histopathological examination for a skin biopsy. (a, b) Hematoxylin and eosin staining shows swelling of the dermis accompanied by mucin deposition, hemorrhagic presentation, lymphocytic and neutrophilic infiltration of the vascular wall and surrounding areas, and a small amount of nuclear dust. (c, d) Immunohistochemistry of immunoglobulin A (IgA) demonstrates IgA deposition within the vascular walls. Panels (b) and (d) depict the same areas at a higher magnification.

Further testing, including urinalysis, revealed mild proteinuria (±) and no hematuria. The patient was negative for myeloperoxidase-antineutrophil cytoplasmic antibody (MPO-ANCA). The serum levels of immunoglobulin G (IgG), immunoglobulin M (IgM), and IgA were within a normal range (1,335, 54, and 164 mg/dL, respectively). Skin biopsy of the rash showed dermal swelling accompanied by mucin deposition, hemorrhagic presentation, lymphocytic and neutrophilic infiltration of the vascular wall and surrounding areas, and a small amount of nuclear dust (Figures 2a, 2b). These findings led to a diagnosis of leukocytoclastic vasculitis. Immunohistochemistry of the skin biopsy demonstrated IgA deposition in the blood vessels (Figures 2c, 2d), confirming a diagnosis of IgA vasculitis. Furosemide has been discontinued since then.

At a 37-day follow-up without systemic treatment but topical anti-inflammatory agents, the patient showed no edema, and the papules had improved. Pain around the bilateral ankle joints was trending toward improvement but remained. Urinary tests revealed no proteinuria or hematuria, and creatinine levels remained stable.

## Discussion

This case report highlights a unique IgA vasculitis initially manifested as bilateral lower extremity edema in an elderly patient. Various conditions can give rise to bilateral lower extremity edema in older individuals, potentially leading to misdiagnosis of rare diseases, such as IgA vasculitis.

A diagnosis of pediatric IgA vasculitis (previously known as Henoch-Schönlein purpura) is confirmed when at least two of four criteria (bowel angina, arthralgia or arthritis, renal involvement, and histopathology) are met, along with the existence of non-thrombocytopenic palpable purpura with lower limb predominance [5]. The attributes of IgAV in mature individuals were prominently demonstrated in an evaluative, retrospective study involving 250 French patients, with a median age standing at 50 years. Initial clinical observations featured palpable purpura (96%), arthritis (61%), and gastrointestinal manifestations (48%) [6]. Kidney dysfunction, as indicated by a creatinine clearance rate less than 50 mL/minute/m^2^, was seen in nearly a third of the patients within a four-month period post presentation [6]. Meanwhile, a smaller case study of adult patients from Northern India revealed higher incidence of arthritis (90%) and abdominal pain in only 10% of adults at presentation [7], which may indicate variations due to genetic and environmental exposures between these countries.

In 2016, a study conducted by Hocevar et al. assessed the applicability of these diagnostic criteria within an adult cohort, revealing a sensitivity of 100% and specificity of 87% [8]. These criteria have been further corroborated by a recent investigation on adult-onset IgA vasculitis, which demonstrated an overall sensitivity of 99% [9]. The patient fulfilled the criteria in the present case through palpable purpura, arthralgia, and a pathological examination. This case study expands our understanding of the atypical manifestations of adult-onset IgA vasculitis, particularly the initial presentation of lower extremity edema. Existing literature infrequently details instances of adult IgA vasculitis initially appearing as lower extremity edema, underscoring the importance for clinicians to maintain a comprehensive differential diagnosis when confronted with this symptom. A documented case report has described an elderly patient with bilateral lower extremity swelling and concurrent gastrointestinal symptoms, further indicating the varied presentation of IgA vasculitis [10].

Frequently, like in this case, a history of upper respiratory tract infections or exposure to specific antigens (e.g., from food, insects, medications, or vaccines) precedes the onset of IgA vasculitis [1]. This pattern suggests that these infections or antigen exposures might trigger IgA vasculitis pathogenesis.

Poor prognostic factors include advanced age at onset (over 50 years), severe renal impairment, macroscopic hematuria, and persistent proteinuria [7]. In this case, we should be careful about the follow-up, given the patient’s advanced age. Despite normal urinalysis findings at the time of diagnosis in this patient, consistent follow-ups are imperative. Adult-onset IgA vasculitis can present with renal involvement, significantly impacting the prognosis even without evident renal abnormalities at initial presentation [4]. Consequently, routine renal function monitoring is recommended, initially bi-weekly for the first-month post-diagnosis and bi-monthly for the following six months to a year [4]. This monitoring schedule takes into account the disease's unpredictable course and the potential for delayed renal complications.

When managing this patient's lower limb pain, we consciously avoided nonsteroidal anti-inflammatory drugs (NSAIDs) due to their adverse effects on exacerbating renal function, a key concern in IgA vasculitis management [4]. Acetaminophen is reported as a safer alternative when necessary [4]. In this case, a prescription for topical loxoprofen was given, and no oral medications were required.

## Conclusions

The present case highlights the importance of considering IgA vasculitis in the differential diagnosis of lower extremity edema. Regular urinalysis and renal function tests are essential, even when initial urinalysis results appear normal. Furthermore, pain management decisions must be made judiciously to prevent potential adverse impacts on the renal function.
